# The Utilization of a Gait Pattern Classification System to Investigate the Effects of Ankle–Foot Orthoses on Gait in Children with Cerebral Palsy

**DOI:** 10.3390/children13050594

**Published:** 2026-04-24

**Authors:** Tobias Goihl, David F. Rusaw, Siri Merete Brændvik, Karin Roeleveld

**Affiliations:** 1 Department of Neuromedicine and Movement Science, Faculty of Medicine and Health, Norwegian University of Science and Technology, 7034 Trondheim, Norway; siri.merete.brandvik@ntnu.no (S.M.B.); karin.roeleveld@ntnu.no (K.R.); 2 Trøndelag Orthopaedic Workshop, TOV, 7030 Trondheim, Norway; 3 School of Health and Welfare, Jönköping University, 553 18 Jönköping, Sweden; david.rusaw@ju.se; 4 Rehabilitation Clinic, St. Olavs University Hospital, 7030 Trondheim, Norway

**Keywords:** spastic cerebral palsy, gait classification, ankle–foot orthoses

## Abstract

**Highlights:**

**What are the main findings?**

- Ankle–foot orthoses (AFOs) influenced swing phase ankle motion and first rocker mechanics in children with drop foot and genu recurvatum gait patterns.
- Flexible/articulated AFOs showed limited correction of stance-phase knee kinematics in genu recurvatum or crouch gait.

**What are the implications of the main findings?**

- Incorporating gait pattern classification when evaluating AFOs may help clarify how functional impairments interact with the mechanical properties of orthoses.
- AFO design should be tailored to the child’s gait pattern to optimize joint mechanics and functional gait outcomes.

**Abstract:**

**Background/Objectives**: Ankle–foot orthoses (AFOs) are commonly used to improve gait in children with cerebral palsy (CP), but their effect on specific gait patterns is underreported. This study evaluates the utilization of the Gait Pattern Classification System for Children with Spastic CP (GaP-CP) to investigate the effects of ankle–foot orthoses on gait kinematics, spatio-temporal parameters and the energy cost of walking. **Methods**: In this retrospective study, 66 ambulatory children with spastic CP underwent 3D gait analysis with and without AFOs or functional electrical stimulation. Gait patterns were classified according to GaP-CP. AFOs were articulated, flexible, or rigid. Thirty-six children also performed a 5 min walk test with gas exchange measurements. Step length, walking speed, and the energy cost of walking were calculated. Gait kinematics were analyzed with statistical nonparametric mapping. Non-parametric statistics were used to investigate orthotic effects for the total group and for each gait pattern. **Results**: Ankle kinematics improved in swing phase and initial contact (10 degrees less plantarflexion, *p* < 0.05) for the total group, dropfoot and genu recurvatum. During the stance phase, reduced knee extension in genu recurvatum (by 3 degrees, *p* < 0.05) and increased knee extension in crouch (by 3 degrees, *p* < 0.05) were observed. Median changes in non-dimensional step length were clinically significant (>0.039, *p* ≤ 0.02, effect size ≥ 0.55) for the total group and the dropfoot, genu recurvatum, and crouch subgroups, while changes in most gait indices, walking speed and the energy cost of walking were not clinically significant. **Conclusions**: The combined use of GaP-CP and kinematic analysis provided new insights into the effects of ankle–foot orthoses on gait. Articulated and flexible orthoses may not have provided adequate support for genu recurvatum and crouch gait, showing a potential value in gait pattern specific orthotic design to optimize gait kinematics.

## 1. Introduction

Ankle–foot orthoses (AFOs) play an important role in managing gait deviations in children with cerebral palsy (CP) [1]. By controlling ankle foot alignment, AFOs directly influence the mechanics of gait, including kinematics and gait indices such as the Gait Profile Score (GPS) or Gait Variable Score (GVS) [2]. However, patient-centered goals often extend beyond mechanical changes and include improved gait function. Here, gait function refers to measurable improvements that reflect how the child performs walking and includes walking speed, step length and the energy cost of walking (ECW) [3,4].

AFOs have been shown to correct dropfoot [5], improve equinus [5,6], genu recurvatum [5,6], jump gait [7], and crouch [5,8] as well as gait indices [2]. The use of AFOs can reduce ECW and increase walking speed and step length [5,6,9]. However, the evidence on the effect of AFOs remains inconsistent, particularly in their influence on gait kinematics and ECW, as shown in a large retrospective study by Ries et al. [10], who highlighted that AFOs can be unreliable in correcting gait indices. The provision of “an AFO” does not necessarily improve gait per se [10]. Studies on the effect of AFOs on ECW have also yielded mixed results. A systematic review by Conner et al. in 2022 [11] reported potential benefits of AFOs in reducing ECW, but the largest retrospective study [12] found this effect only in children with quadriplegia. Some studies have reported significant reductions in energy expenditure [13,14,15], while others found no significant change or even an increase [16,17,18] in preferred walking speed.

Some researchers have given AFO-design suggestions for specific gait patterns [1,19,20]. Others recommend rigid AFOs for all children with spastic CP who display abnormal shank alignment in stance [21], in part to protect the structural integrity of the foot. However, it has not been possible to link improvements in gait kinematics to specific AFO designs [22], and this uncertainty is reflected in several national guidelines, which do not provide an overview of gait deviations with matching AFO design recommendations [23,24,25]. Registered data from Sweden [26] and a national survey from Norway [27] show frequent use of flexible and jointed AFOs and that the choice of AFO design is not dependent on the functional impairments of the child [28] or gait pattern [27]. Even functional electrical stimulation (FES) orthoses, though not classified as AFO [29], are increasingly used as alternatives to traditional AFOs for children with CP [11,30].

Numerous gait classification systems (GCS) were developed over time to provide a common language for describing and comparing typical gait deviations seen in children with CP [31]. The qualitative GCS are based on clinical observations and expertise and commonly capture distinct patterns at mid-stance but do not cover the complete gait cycle or the full spectrum of deviations [32]. They may be intuitive, but can be subjective and difficult to standardize. These limitations reduce the clinical implementation of GCS and complicate the effort to link specific gait patterns to optimal AFO prescriptions. The Gait Pattern Classification System for Children with Spastic Cerebral Palsy (GaP-CP [32]) was developed to address these shortcomings: it classifies uni- and bilateral spastic CP across seven clinically relevant gait patterns (mild deviations, dropfoot, genu recurvatum, true equinus, jump gait, apparent equinus, and crouch gait). It is validated with data-driven rules, applied to the entire gait cycle and shows high content and construct validity [32].

GaP-CP offers a promising approach to reduce variability of outcome measures by enabling a more nuanced, gait-pattern-specific analysis of AFO effects on both gait mechanics and gait function. This could support more personalized and effective AFO prescriptions. Everaert et al. [2] demonstrated that GaP-CP could be used to evaluate the effect of rigid AFOs on specific gait patterns and found significant improvements in the total group and in crouch gait. However, the effect of commonly used flexible or jointed AFO-designs has not been investigated with this method.

The aim of the current study is to evaluate whether using GaP-CP can improve the identification of AFO effects on both mechanical (gait kinematics and gait indices) and functional outcomes (walking speed, step length, ECW) in children with spastic CP. We hypothesize that the effect of AFOs will differ across GaP-CP gait patterns, and that GaP-CP stratification will reduce variability and reveal pattern-specific AFO effects that are not apparent in the total group.

## 2. Materials and Methods

### 2.1. Participants

This exploratory, retrospective study is based on data from a randomized controlled trial (N = 36) [33] and clinical data from the gait laboratory at St. Olavs Hospital, Trondheim (N = 30), both collected using the same standardized procedure for data capture. Because GaP-CP categorizes gait into seven distinct patterns, a sufficiently large sample is required for subgroup analyses. Therefore, all eligible datasets available within our research facility were combined. It is acknowledged that participants in these two groups may differ and that the clinical dataset may include more severely affected children. However, this variability is expected to be reduced by the use of GaP-CP. In both settings, children were given ample time to rest and to become accustomed to the different walking conditions, so that observed differences in gait between conditions are expected to stem from the use of AFOs.

Inclusion criteria were children and adolescents aged 4–18 years with uni- or bilateral spastic CP who use AFOs, classified as level I-III according to the Gross Motor Function Classification System (GMFCS) [34]. The included AFO types were rigid (no ankle movement), articulated (mechanical ankle joint), flexible (such as ToeOff^®^, Allard, Helsingborg, Sweden) and FES. All participants who fulfilled these criteria with valid data from the 3-dimensional gait analysis (3DGA) were included. Ethical approval was granted by the regional Norwegian ethics committee for medical and health research in Norway (Ref. 2013/1195, 22 January 2014, REK nord for the randomized controlled trial, extended 2 April 2025; Ref. 2010/1991, 20 September REK sør-øst B and Ref. 2017/2066, 9 September 2024 REK midt for use of clinical data from the gait laboratory).

Written informed consent was provided.

### 2.2. Procedure and Equipment

#### 2.2.1. 3D Gait Analysis

For 60 participants, 3DGA was conducted using Vicon Motion Capture systems (Vicon, Oxford Metrics, Oxford, UK) and for 6 participants, the Qualisys Motion Capture system was used (Qualisys AB, Gothenburg, Sweden). Seven to 10 optical cameras with a sampling frequency of 150–200 Hz. Sixteen reflexive markers were placed on anatomical landmarks on the lower limbs according to the Vicon Plug-in-Gait model [35] or 28 markers according to the Qualisys CAST [36]. Proprietary software from Vicon (Nexus) and Qualisys (QTM) were used to define gait cycles and calculate joint angles and spatiotemporal gait parameters. All data from 3DGA were imported as c3d-files into a customized Matlab program (R2020b, MathWorks, Inc., Natick, MA, USA), written using the Biomechanical Toolkit (Btk Development Core Team, Version 0.3.0) for data inspection and further processing. Every participant had recordings of at least three trials with one to three gait cycles for each condition. Visual quality control of gait traces was performed according to pre-defined criteria by the same researcher with more than 10 years of experience with 3DGA (TG). Gait cycles with outliers (deviating more than two standard deviations from the average kinematic curve), artifacts (identified from a comparison of individual traces to the averaged kinematic graph) or signs of inaccurate marker position (discrepancies between kinematic data and clinical assessment) were excluded [2]. From each participant, the most affected side was determined by TG from visual inspection of ankle- and knee kinematics in the sagittal plane as the side that showed most deviation from our normative dataset of 24 typically developed children (mean age 11.1 years). Kinematic data from the most affected side were used to calculate the averaged gait cycles for conditions _without_AFO and condition _with_AFO. Kinetic analysis was not performed because 45% of participants had incomplete force plate data. The first author (TG) performed the GaP-CP gait classification based on kinematic data. TG received training in the GaP-CP classification procedure from its creator [32] over several online meetings, and cases which were unclear were discussed. TG and SB collaborated on the implementation of GaP-CP.

The use of 3DGA data from different motion capture systems using two different marker sets has been reported previously [37], and comparisons between different marker models find good reliability of sagittal plane kinematics [38], as most variability within and between marker models has been reported from transverse plane kinematics, which was only used for two out of nine variables on which the GPS is based on. The GaP-CP classification is based on the pelvis, hip, knee and ankle data across the entire gait cycle and is not expected to be affected by minor differences between datasets. Careful visual inspection of the gait traces did not reveal any apparent systematic differences between datasets. Moreover, differences in outcome variables between the AFO and non-AFO conditions are expected to be even less influenced by the motion capture system and biomechanical model, as any systematic offsets would be present in both conditions.

#### 2.2.2. Energy Cost of Walking

To calculate ECW, oxygen uptake (VO2) and carbon dioxide production (VCO2) were measured with concurrent gas exchange measurements using Metamax, version II or IIIb (Cortex Biophysik GmbH, Leipzig, Germany). The mobile gas analyzer was calibrated according to the manufacturers’ instructions and carried by the participants in a harness. A member of staff walked behind the participants with a distance-measuring wheel to record meters walked.

#### 2.2.3. Procedure

Thirty-six participants were assessed with both ECW and 3DGA. An additional thirty participants were assessed with 3DGA only. ECW (J/kg/m) was obtained using a 5 min walk test (5MWT) protocol [39] at preferred walking speed along a 45 m pathway, first in condition _without_AFO, then in condition _with_AFO (both with shoes) with a resting period in between. Gait speed was calculated during the last two minutes of each 5MWT and from this period, VO2 and VCO2 were averaged across 1 min steady state.

**Figure 1:**
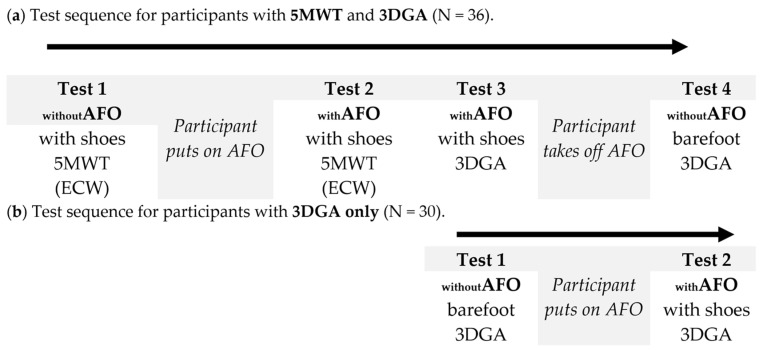
Illustration of test sequences for participants performing both the 5MWT and 3DGA (**a**) and 3DGA only, (**b**) with information on footwear (shoes/barefoot and AFO (_without_AFO/_with_AFO) conditions. 3DGA = 3-dimensional gait analysis; 5MWT = 5 min walk test; ECW = energy cost of walking.

For the 3DGA, all participants walked at self-selected walking speed on a walkway for at least 7 m for a minimum of six trials per condition. Participants with ECW conducted 3DGA first in the condition with shoes with AFO (_with_AFO) and then in the condition of being barefoot (_without_AFO). For participants without ECW, 3DGA was done first in condition _without_AFO and then in condition _with_AFO (Figure 1). The sequence of conditions was chosen to minimize the number of times participants had to take off/put on their AFO and this lack of randomization is unlikely to overestimate the effect of AFOs on ECW as this condition was always measured last. Resting periods between tests and conditions were adjusted to each participant to prevent fatigue and sustain motivation.

AFO designs were identified using the study protocol of Ref. [33] or images from 3DGA. GaP-CP was applied based on the inspection of the averaged traces for each participant for condition _without_AFO [2].

### 2.3. Outcome Variables

Gait kinematics and gait indices were used to describe the mechanics of gait. Non-dimensional step length, non-dimensional walking speed and ECW were used to describe gait function.

Gait profile score (GPS [40]) was calculated from the gait kinematics of the most affected side. For GPS, excellent reliability has been reported for children with CP [41]. The GPS was derived from nine kinematic gait variable scores (GVS [40], from pelvis anterior/posterior tilt, obliquity, internal/external rotation; hip flexion extension, ab-/adduction, internal/external rotation; knee flexion/extension; ankle dorsi-/plantarflexion; and foot progression). Each score was calculated as the root mean square difference between the participants’ joint kinematics and that from our normative data set. Individual, sagittal plane GVSs for hip, knee and ankle are also reported. All scores are given in degrees and a reduction in value indicates fewer differences from normative data [2,40].

From 3DGA, the non-dimensional step length (ND-SL) and non-dimensional walking speed (ND-WS3DGA) were calculated using the following equations [42]:

ND−SL=(step length(m))⁄(Leg length(m))

〖ND−WS〗_3DGA=〖speed〗_3DGA(m⁄s)/√((9.81⁄(s^2 x leg length(m))))

From 5MWT, the non-dimensional walking speed (ND-WS5MWT) was calculated according to the same method as above. ECW (J/kg/m) was calculated according to the following equations [39]:

ECW=(4.960∗VO2/VCO2)+16.040)∗(VO2/body weight)/walking speed [m/min]

### 2.4. Statistical Analysis

Statistical analyses were performed for all participants (total group) and for each subgroup of Gap-CP with at least 10 participants. Because most data were not normally distributed (Shapiro–Wilk test < 0.05), non-parametric statistics were used. Significance was set to 0.05 and values for minimal clinically important differences (MCID) between conditions _without_AFO/_with_AFO are reported for variables with existing reference values [2].

To explore the difference in kinematics between conditions, statistical non-parametric mapping (SnPM) for the gait cycle, normalized kinematics was performed for the hip, knee, and ankle joint in the sagittal plane in both conditions for total and sub-groups [2] in Matlab (R2020b). SnPM performed with 1000 iterations and α = 0.05 (SPM-SPM1d version 0.4.7, www.spm1d.org). The analyses were first performed on a three-component vector level (two-sample Hotelling’s T2 test). Then, a joint-level post hoc (paired *t*-test) comparison was conducted for the hip, knee and ankle joints [2]. We applied the same SnPM statistics as the only previous GaP-CP study evaluating the effect of AFOs [2], even though this approach may underestimate the true differences between conditions. As no MCID is defined for this measure, thresholds for clinical relevance were implemented: a cluster duration of min. 3% length of the gait cycle and difference in mean values above the pre-defined standard error of measurement (SEM) [2,38]. Because this study is considered exploratory, no formal multiple-testing correction was applied, as such correction might have obscured potentially meaningful patterns in the data.

For gait indices, step length, walking speed and ECW, median values and median change between conditions with inter-quartile range (IQR) are reported and the paired Wilcoxon signed-rank test was used to investigate the AFO effect (IBM SPSS Statistics for Windows, version 29, IBM Corp., Armonk, NY, USA).

To investigate the effect of AFO on ECW, a subset of 36 participants was analyzed, using the same Gap-CP groups even though one group had fewer than 10 participants. The change between conditions in ECW was compared to the smallest detectable difference for ECW, which is reported to be 6.8% [39]. Correlation between changes in ECW and ND-WS5MWT was explored with Kendal’s tau.

## 3. Results

### 3.1. Participants

Sixty-six participants aged 5–18 years (median 9), GMFCS I-II, were included (Table 1). Eighteen were classified with dropfoot, 21 with genu recurvatum and 12 with crouch gait. The remaining 15 participants were classified with the following gait patterns: mild deviations (N = 2), true equinus (N = 2), jump gait (N = 7), apparent equinus (N = 3), or not classifiable (N = 1).

### 3.2. AFO Designs

Participants used their own AFOs, provided by their local orthotist. Included AFO-designs and their use for different gait patterns are described in Table 2. Articulated or flexible AFOs and FES were used by 97% of participants, while rigid AFOs were only used by 3%. Supplementary Table S1 contains information for each participant on gait pattern, the ankle range of motion and AFO properties. Because of local ethical regulations, we are unable to publish a complete set of raw data.

### 3.3. AFO-Effects on Mechanics of Gait; Gait Kinematics and Gait Indices

**Figure 2:**
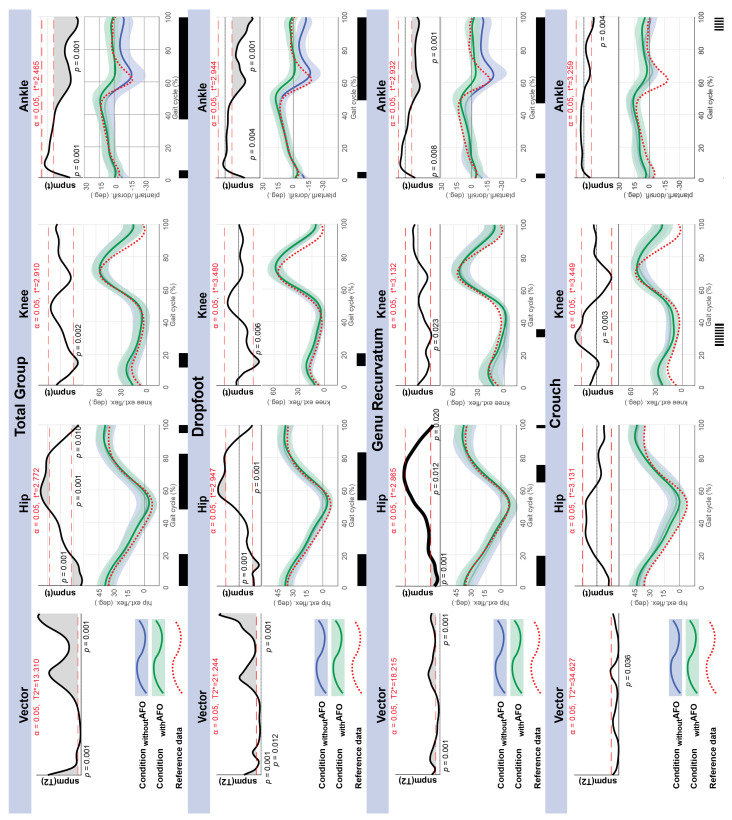
SnPM vector analyses and post hoc analysis for hip, knee, and ankle angles in the sagittal plane across 100% of the gait cycle, comparing kinematics in conditions _without_AFO with condition _with_AFO, for the total group and each GaP-CP subgroup. SnPM analyses are presented with the black solid lines at the top of the graphs. The red dashed horizontal lines represent the critical thresholds (T2*/t*) corresponding to α = 0.05. In the middle of each graph, kinematic traces (mean and 1SD) in blue represent condition _without_AFO, traces in green represent condition _with_AFO, red traces represent reference data from typically developed children. Black bars in the bottom of the graphs visualize periods of the gait cycle where difference between conditions is significant (*p* < 0.05) and exceeds SEM for at least 3% of the gait cycle. For crouch gait, results for post hoc analysis are shown with striped bars, because the vector analysis was only significant for a very short duration at the beginning of swing phase.

Gait kinematics for the total group and subgroups (dropfoot, genu recurvatum, crouch) were analyzed by SnPM and the results are shown in Figure 2. Analysis of the sagittal plane kinematic vector of the total group and subgroups showed statistically significant differences between conditions _without_AFO/_with_AFO (*p* ≤ 0.036) which extended for most of the gait cycle, except for crouch gait, where the vector had only one short cluster (4.5%) at the beginning of swing. The post hoc analysis of the single joints revealed significant differences between conditions (*p* ≤ 0.008) and extended for at least 3% of the gait cycle and exceeded SEM thresholds. SnPM illustrates where in the gait cycle differences between conditions occur. The averaged traces show the direction of the differences between conditions _without_AFO and _with_AFO (Figure 2). At the level of the ankle joint, use of AFOs increases dorsiflexion at initial contact and reduced plantarflexion in swing for the total group and the dropfoot and genu recurvatum subgroups. In late stance (50–60% of the gait cycle), the use of AFOs reduced plantarflexion or push-off movement. At mid-stance (30% of the gait cycle), there were no significant AFO effects for any of the groups at the ankle joint. For the knee, stance-phase extension was reduced in the genu recurvatum subgroup and stance-phase flexion was reduced for the crouch gait subgroup.

Of all gait indices, only the GVS ankle showed significant reductions (*p* ≤ 0.001) for condition AFO in median value for both, total group (−1.93°) and genu recurvatum (−3.17°), both of which were above the MCID threshold of 1.5° (Table 3). GPS, GVS knee, and GVS hip did not show any significant AFO effect for the total or any of the subgroups.

### 3.4. AFO-Effects on Gait Function: Step Length, Walking Speed and Energy Cost of Walking

The effects of AFO on gait function are shown in Table 4. ND-SL increased significantly (*p* ≤ 0.02) when using AFOs for the total group and all subgroups, exceeding MCID of 0.039. The median change in ND-SL in the genu recurvatum (0.1) and crouch gait (0.1) subgroups was larger than that in the dropfoot subgroup (0.045). Changes in ND-WS_3DGA_ did not reach MCID for the total or any subgroup.

**Figure 3:**
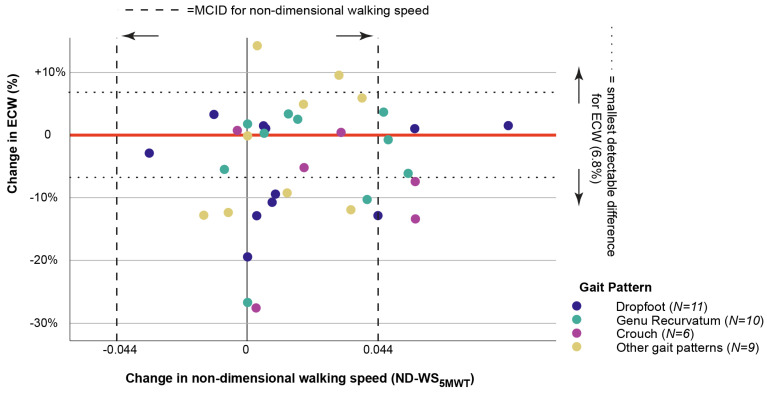
Changes in the energy cost of walking vs. changes in the walking speed in condition. Each participant is plotted according to their percentage change in ECW and change in ND walking speed from the 5 min walk test (ND-WS_5MWT_) in condition _with_AFO when compared to _without_AFO. The change in ECW is given in percent. Smallest detectable difference for ECW of 6.8% is highlighted with two horizontal, dotted lines. The change in ND-WS_5MWT_ is given in absolute numbers. The threshold for minimal clinically important difference (MCID) of ±0.044 is highlighted with two vertical, dashed lines. Participants that lie to the right of the dashed 0.044 line increased ND-WS_5MWT_ in condition _with_AFO above the MCID. Participants that lie below the dotted −6.8% line have decreased their ECW by more than the smallest detectable difference in condition _with_AFO.

Of the 66 included participants, 36 had undergone the 5MWT to evaluate ECW. Median change in ECW did not reach the threshold for minimum detectable difference for the total group or any subgroup. Changes in non-dimensional walking speed during 5MWT (ND-WS_5MWT_) and ECW are not correlated (*p* = 0.445). The three participants with the largest reduction in ECW show very little change in walking speed (less than half of MCID, Figure 3). The two participants who show the greatest increase or decrease in walking speed in condition AFO have almost no change in ECW. The observed lack of correlation is also true for the subgroups dropfoot, genu recurvatum and crouch gait (*p* > 0.3).

## 4. Discussion

In this study, we examined the combined use of GaP-CP and performed kinematic analysis to better understand how AFOs influence gait mechanics and functional outcomes in children with CP. The results show that AFOs can produce measurable changes in sagittal-plane kinematics for the total group and for the dropfoot and genu recurvatum subgroups. Functional outcomes showed a consistent increase in step length, while changes in walking speed and ECW were limited and did not become more evident in subgroups. Taken together, these findings indicate that GaP-CP can help structure the interpretation of AFO effects according to gait patterns.

The results offer important insights into the benefits of using GaP-CP for the two stages of AFO evaluation: their direct impact on gait mechanics and their indirect impact on gait function, including parameters that matter to patients, namely walking speed and ECW. It should be recognized that the estimated AFO effects on gait kinematics, step length, walking speed from 3DGA and gait indices reflect differences between barefoot gait and AFO-shoe combination. However, the effects on ECW and walking speed from 5MWT are based on comparisons between shoed gait and AFO-shoe combination.

### 4.1. AFO Design

Nearly all participants in this study (97%) had been provided with AFOs that allowed for varying degrees of ankle movement: articulated and flexible AFOs or FES. This contrasts with Everaert et al. [2], where rigid AFOs dominated. In part, the use of rigid AFO designs may be explained by the participants, which were more affected in the Everaert study, which included 11.8% participants at GMFCS level III. However, the preference for articulated and flexible designs in our study, particularly in children with genu recurvatum and crouch gait, is noteworthy. While the literature recommends rigid AFOs to optimize gait and protect the musculoskeletal system [20,22,43], articulated AFOs have been used with some success for crouch gait [13] and the use of flexible designs reflects preferences of some clinicians and patients for lightweight orthoses that allow for more movement [27]. Because AFO designs vary in function, assessing gait-pattern-specific effects for each design is challenging and requires larger samples for robust conclusion.

### 4.2. AFO Effect on Mechanics of Gait; Kinematics and Gait Indices

The utilization of GaP-CP in this study provided insights into the joint-specific effects of AFOs on gait mechanics. AFOs reduced dropfoot in the swing phase, improved pre-positioning for initial contact and established the 1st rocker for the total group and for the dropfoot and genu recurvatum subgroups. The use of AFOs also reduced push-off movement in the late stance. These results are like those reported by Everaert et al., although they did not find an improved 1st rocker. This lack of the 1st rocker might be related to the rigid AFO design used in their study, which does not allow for the plantarflexion movement required at this phase of gait. The reduction in push-off is a consequence of using AFO designs, which limit plantarflexion. The use of spring-loaded joints or more effective energy return in flexible designs may have the potential to improve this important gait feature. Unlike Everaert et al., we did not observe an AFO effect in genu recurvatum and crouch gait during stance-phase dorsiflexion, likely due to the use of articulated and flexible AFOs, which lacked the rigidity to fully control ankle movement during this phase of gait.

At the knee joint, AFOs increase stance-phase knee flexion in genu recurvatum, and decrease stance-phase knee flexion in crouch gait. However, these changes were not significant for the entire stance phase. The limited knee corrections may be related to the use of non-rigid AFOs, which did not sufficiently influence knee mechanics. The mechanical effectiveness of rigid AFOs in correcting crouch gait has been reported before [44,45], although Kerkum et al. [13] have shown that spring-loaded, articulated AFOs can correct gait kinematics in crouch gait as well. Most children in our study used flexible AFOs or articulated AFOs with open dorsiflexion, which are designs that do not allow for adjustments of ankle stiffness to prevent excessive dorsiflexion. The use of AFOs with open dorsiflexion for genu recurvatum with short gastrocnemius is not in accordance with recommendations [20,43] but was described as a common choice in a recent survey on AFO provision practice in Norway [27]. The use of this type of AFO for crouch gait is not in accordance with recommendations [19,20] and was not described as a choice in the national survey [27]. This raises the possibility that gait deviations may have been assessed differently by clinicians, highlighting the potential value of incorporating more objective and systematic assessment routines in clinical practice.

The only significant AFO-related improvement in gait indices was observed at the ankle (GVS-ankle) for the total group and the genu recurvatum subgroup. The low sensitivity of gait indices in detecting AFO effects has been reported [11], but may also reflect the suboptimal correction of kinematics by articulated or flexible AFOs achieved in our study. Gait indices are useful for quantifying gait pathology, but they cannot inform AFO prescriptions and may lack the sensitivity to detect clinically significant AFO effects [2,11,46]. One potential benefit of analyzing the effect of AFOs on each gait pattern separately lies in the different requirements and functions of AFOs. As illustrated for the knee joint, the expected correction runs in opposite directions for genu recurvatum and crouch gait. Applying GaP-CP may provide an explanation for why GVS-knee and overall GPS were not affected by AFO, as group-wise analysis of gait kinematics indicated that AFO effects were mostly related to the ankle joint in dropfoot and genu recurvatum.

### 4.3. AFO Effect on Gait Function; Step Length, Walking Speed and the Energy Cost of Walking

Functional outcomes are often more critical to the patients than purely mechanical corrections [11]. Using GaP-CP, we found median that changes in ND-SL were twice as large in genu recurvatum and crouch gait compared to dropfoot (0.1 vs. 0.045), which may indicate a gait-pattern specific AFO effect. Both Everaert and Ries reported AFO-related increases in ND-SL. In contrast to these studies, we did not find changes in ND-WS_3DGA_ that reached clinical significance.

ECW is perhaps the most important functional outcome for ambulatory patients, as it potentially impacts daily activity directly [33]. Among the subset of 36 participants, we did not observe a reduction in ECW that reached the detectable threshold of 6.8% for the total group or any subgroups. The utilization of GaP-CP showed a difference between gait patterns as both median change and interquartile range of ECW was largest in crouch gait. Large variability in ECW mirrors findings from previous studies [11,12], where AFO effects on gait efficiency varied widely. While some studies have demonstrated the potential for AFOs to reduce ECW by 9–33% [13,47,48], Betancourt et al. [18] concluded in their systematic review that the literature is inconsistent on whether AFOs increase or decrease the metabolic cost. Factors that may contribute to differences between studies may include differences in study design (overground [13] vs. treadmill walking [15]), methodology (various direct and indirect measures of energy expenditure have been used [11]) and differences in AFO provision practice. A survey from 2024 has reported that choices for AFO designs for each gait pattern vary among orthotists [27].

### 4.4. Strengths and Limitations

The strength of this study is the use of GaP-CP to classify participants by gait pattern, which may help reveal subgroup-specific AFO effects that could be overlooked in analyses of the total group. However, as most participants used articulated or flexible AFOs or FES, the generalizability of our findings is limited with regard to other AFO designs. The treatment goals of the AFOs were not reported. Therefore, some AFOs included may not have been prescribed for gait optimization alone but may have been designed to accommodate the desire for mobility, patient preferences, or ease of use [9,27]. To improve transparency, AFO properties and ankle range of motion is provided for each participant in Supplementary Table S1. Because the 3DGA analyses compare barefoot gait with the AFO–shoe condition, the resulting effects reflect the combined influence of the AFO and footwear, a limitation common in AFO research [49]. A potential source of error in this study is the use of data from two different motion capture systems. However, because sagittal-plane kinematics show good reliability across marker models [38], the reported AFO effects and the GaP-CP classifications are not expected to be affected by this. There may be some uncertainty related to the calculation of the GPS, as it includes two transverse plane variables, but we did not find any significant differences between conditions and can therefore exclude a type I error. Despite these limitations, this approach demonstrates the feasibility of combining data from multiple gait laboratories to increase the sample size and enable more advanced data analyses. Nevertheless, larger studies that include a broader range of AFO designs and gait patterns are needed to build on these findings. Examining gait pattern-specific effects of AFOs may improve orthotic management by more systematically linking AFO properties to gait deviations, thereby informing the development of more detailed prescription guidelines and evaluation criteria.

## 5. Conclusions

This study suggests that combining GaP-CP with kinematic analysis can provide additional insight into how AFOs influence gait mechanics in children with CP. Articulated and flexible AFOs produced modest improvements in ankle kinematics in dropfoot and genu recurvatum, but these changes did not consistently translate into meaningful corrections at the knee or ankle during stance in genu recurvatum or crouch gait. Functional effects were limited: the step length improved, whereas the walking speed and ECW did not. The small magnitude of the kinematic changes likely explains the absence of significant ECW reductions, which remains a key clinical concern. Overall, the findings highlight the need to further investigate which AFO design features are the most appropriate for specific gait patterns, as such pattern-specific insights may support more targeted and effective orthotic management.

## Figures and Tables

**Table 1:** Participants’ characteristics for the total group and for each gait pattern with N > 10.

		Total Group	Dropfoot	Genu Recurvatum *	Crouch Gait
			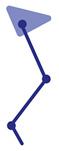	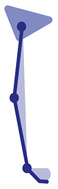	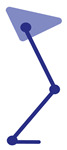
GaP-CP classification rules for		Knee	Increased FL at TSw and IC	Full knee Ext or HE in stance	Increased FL in stance
		Ankle	Increased PFL in swing	Decreased DFL in midstance	Increased DFL in stance
		**N = 66**	**N = 18**	**N = 21**	**N = 12**
Age, years	median (IQR)	9 [7.5;12.6]	9.4 [8.8;12.7]	8.9 [7.2;13.7]	10.0 [7.6;15.6]
Height, m	median (IQR)	1.38 [1.26;1.54]	1.48 [1.35;1.57]	1.45 [1.24;1.54]	1.46 [1.20;1.64]
Weight, kg	median (IQR)	32.3 [25.4;49.9]	42.8 [29.6;54.0]	35.5 [26.7;48.3]	38.1 [23.5;63.6]
Girls	(%)	41%	28%	57.1%	33%
GMFCS I	in %	62.1%	77.8%	57.1	16.7%
GMFCS II	in %	37.9%	22.2%	42.9%	83.3%
Unilateral	in %	72.7%	44.4%	57.1%	42%

* According to GaP-CP, limbs with increased knee extension in stance with concurrent plantarflexion were classified as genu recurvatum and not as equinus gait. GaP-CP = Gait Pattern Classification system for children with spastic CP; FL = flexion; TSw = terminal swing; IC = initial contact; Ext = extension; HE = hyperextension; DFL = dorsiflexion; IQR = 25th and 75th quartiles; GMFCS = Gross Motor Function Classification System.

**Table 2:** Proportion of AFO designs for total group and each gait pattern, given in %.

		FES	Flexible	Articulated *	Articulated **	Rigid AFO	Total
		N = 3	N = 30	N = 27	N = 4	N = 2	N = 66
**Gait Pattern**	**N**	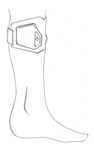	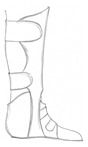	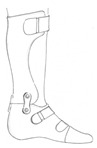	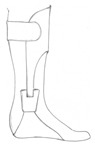	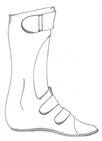	
Total group	66	4.5%	45.5%	40.9%	6.1%	3%	100%
Mild deviations	2	50%	50%	-	-	-	100%
Dropfoot	18	5.6%	72.2%	22.2%	-	-	100%
Genu Recurvatum	21	4.8%	38.1%	57.1%	-	-	100%
True Equinus	2	-	-	50%	50%	-	100%
Jump Gait	7	-	14.3%	57.1%	28.6%	-	100%
Apparent Equinus	3	-	-	66.7%	-	33.3%	100%
Crouch gait	12	-	50%	41.7%	8.3	8.3%	100%
Not classifiable	1	-	100%	-	-	-	100%

FES = functional electrical stimulation; * = Free dorsiflexion. plantarflexion stop; ** = spring loaded joint.

**Table 3:** Gait indices for total group and subgroups of dropfoot, genu recurvatum and crouch gait: Gait Profile Score (GPS) and Gait Variable Score (GVS) for ankle, knee and hip for condition _without_AFO and condition _with_AFO with interquartile range. Median change between conditions is provided with the level of significance (*p*-value). Minimal clinically important difference (MCID) is given for each variable.

		Total Group	Dropfoot	Genu Recurvatum	Crouch Gait	MCID
		**N = 66**	**N = 18**	**N = 21**	**N = 12**	
GPS (°)	_without_AFO median (IQR)	10.77 [9.01;12.11]	9.84 [8.58;11.00]	10.75 [8.95;11.59]	11.21 [9.70;12.55]	
	_with_AFO median (IQR)	10.59 [8.96;11.82]	9.86 [8.63;10.77]	9.78 [8.27;11.48]	11.38 [9.45;12.82]	
	Median change	−0.13 [−1.08;0.50]	+0.14 [−1.08;0.65]	−0.22 [−0.94;0.48]	−0.13 [−0.86;0.89]	1.6°
	*p*-value	0.078	0.98	0.19	0.81	
	Effect size	0.22	−0.01	0.28	0.07	
GVS (°) ankle	_without_AFO median (IQR)	9.01 [6.70;13.39]	8.23 [6.85;11.72]	9.50 [6.77;14.32]	7.75 [6.69;9.97]	
	_with_AFO median (IQR)	6.57 [5.21;9.35]	6.81 [6.05;7.99]	5.83 [4.27;8.32]	9.16 [6.71;11.49]	
	Median change	**−1.93 [−7.45;1.08]**	−1.12 [−6.37;1.00]	**−3.17 [−7.78;−1.35**]	**1.57 [−0.09;2.34]**	1.5°
	*p*-value	<0.001 *	0.142	0.001 *	0.158	
	Effect size	0.42	0.33	0.70	−0.41	
GVS (°) knee	_without_AFO median (IQR)	16.72 [12.95;19.61]	15.21 [12.64;16.74]	14.35 [9.49;18.03]	18.07 [16.26;22.72]	
	_with_AFO median (IQR)	16.42 [12.86;19.56]	15.20 [12.86;17.44]	13.28 [10.31;17.78]	17.41 [15.22;25.15]	
	Median change	−0.20 [−1.67;2.42]	−0.06 [−0.97;3.17]	−0.15 [−2.51;1.83]	−0.23 [−1.72;2.61]	3.4°
	*p*-value	0.685	0.306	0.614	0.937	
	Effect size	−0.05	−0.24	0.11	0.02	
GVS (°) hip	_without_AFO median (IQR)	11.33 [8.41;15.14]	11.19 [7.71;13.06]	10.68 [7.23;13.60]	13.36 [10.51;18.34]	
	_with_AFO median (IQR)	10.98 [9.07;15.49]	10.03 [7.61;14.92]	10.79 [8.45;14.94]	14.79 [11.03;19.61]	
	Median change	0.69 [−0.88;2.46]	0.19 [−0.88;2.06]	0.69 [−1.07;2.38]	0.20 [−1.68;2.35]	1.5°
	*p*-value	0.073	0.5	0.274	0.638	
	Effect size	−0.22	−0.16	−0.24	−0.14	

IQR = 25th and 75th quartiles; Effect size = Z/√N. **Bold** = median change above minimal clinically important difference; * statistically significant with α = 0.05.

**Table 4:** Gait function (step length, walking speed, energy cost of walking) in condition _without_AFO and _with_AFO for total group and subgroups of dropfoot, genu recurvatum and crouch gait and the median change between conditions. (**a**) Data for step length and walking speed of all participants. (**b**) Data for energy cost of walking for subset of 36 participants.

			Total Group	Dropfoot	Genu Recurvatum	Crouch	
(a)			N = 66	N = 18	N = 21	N = 12	MCID
	ND SL	_without_AFO median [IQR]	0.7 [0.61;0.77]	0.66 [0.60;0.78]	0.72 [0.66;0.79]	0.62 [0.56;0.69]	
		_with_AFO median [IQR]	0.78 [0.68;0.85]	0.74 [0.64;0.85]	0.80 [0.72;0.90]	0.7 [0.62;0.79]	
		Median change [IQR]	**0.08 [0.28;0.13]**	**0.045 [−0.08;0.10]**	**0.1 [0.05;0.14]**	**0.1 [0.06;0.15]**	0.039
		*p*-value	<0.001 *	0.02 *	<0.001 *	0.004 *	
		Effect size	0.75	0.55	0.80	0.84	
	ND WS_3DGA_	_without_AFO median [IQR]	0.39 [0.33;0.43]	0.36 [0.3;0.41]	0.36 [0.35;0.43]	0.32 [0.26;038]	
		_with_AFO median [IQR]	0.42 [0.35;0.46]	0.38 [0.34;0.44]	0.42 [0.36;0.47]	0.41 [0.32;0.47]	
		Median change [IQR]	0.03 [−0.01;0.07]	0.03 [−0.01;0.056]	0.03 [−0.02;0.05]	0.03 [−0.08; 0.1]	0.044
		*p*-value	<0.001	0.163	0.103	0.03	
		Effect size	0.45	0.33	0.36	0.63	
**(b)**			**N = 36**	**N = 11**	**N = 10**	**N = 6**	**SDD**
	ECW (j/kg/m)	_without_AFO median(IQR)	5.04 [4.10;7.59]	5 [4.04;5.64]	4.64 [3.35;6.07]	8.3 [4.53;12.85]	
		_with_AFO median (IQR)	4.68 [4.03;7.06]	4.4 [4.02;5.48]	4.57 [3.28;5.86]	7.8 [4.49;11.08]	
		Median change [IQR]	−0.12 [−0.64;0.08]	−0.21 [−0.6;0.06]	−0.01 [−0.32;0.13]	−0.33 [−2.3;0.35]	
		Median change in % [IQR]	−1.8% [−11.6;1.7]	−2.9% [−12.8;1.5]	−0.2% [−7.1;2.7]	−6.3% [−16.9;0.5]	6.8%
		*p*-value	0.02	0.109	0.386	0.116	
		Effect size	−0.39	−0.48	0.12	−0.64	

ND SL = non-dimensional step length; ND-WS_3DGA_ = non-dimensional walking speed during 3D gait analysis; ECW = Energy Cost of Walking; IQR = 25th and 75th quartiles; for ECW, the median change is also given in % because the reference value SDD is given in %; Median change = median of changes between conditions for all subjects; MCID = minimum clinically important difference; SDD = smallest detectible difference; Effect size = Z/√N. **Bold** = median change above minimal clinically important difference; * statistically significant with α = 0.05.

## Data Availability

The data are not publicly available due to local ethical regulations and cannot be shared.

## Supplementary Materials

The following supporting information can be downloaded at: https://www.mdpi.com/article/10.3390/children13050594/s1, Table S1: Clinical information and AFO properties.

## Abbreviations

The following abbreviations are used in this manuscript:

AFO/AFOs	Ankle–Foot Orthosis/Orthoses
GaP-CP	Gait Pattern Classification System for Children with Spastic Cerebral Palsy
3D	Three-dimensional
5MWT	5 min Walk Test
CP	Cerebral Palsy
GPS	Gait Profile Score
GVS	Gait Variable Score
ECW	Energy Cost of Walking
FES	Functional Electrical Stimulation
GMFCS	Gross Motor Function Classification System
3DGA	Three-Dimensional Gait Analysis
VO2	Volume of Oxygen uptake
VCO2	Volume of Carbon dioxide production
ND-SL	Non-dimensional step length
ND-WS_3DGA	Non-dimensional walking speed during 3D gait analysis
ND-WS_5MWT	Non-dimensional walking speed during 5 min walk test
MCID	Minimal clinically important difference
SnPM	Statistical non-parametric mapping
SEM	Standard error of measurement
IQR	Interquartile range
SDD	Smallest detectable difference
