# Supplementary material for: The Utilization of a Gait Pattern Classification System to Investigate the Effects of Ankle–Foot Orthoses on Gait in Children with Cerebral Palsy
Source: Children (Basel). 2026 Apr 24;13(5):594. doi: 10.3390/children13050594 (PMC13204945; doi:10.3390/children13050594)
Supplement: Supplementary file 1 [file children-13-00594-s001.zip › children-4223190-supplementary.pdf]

Supplementary Table S1: Clinical information and AFO properties

Each participant is described with their gait pattern (classified according to GAP-CP, Papageorgiou et.al,2025) age, CP-distribution, GMFCS (Gross Motor Classification System) and ankle range of motion (ROM) of the most affected side with the knee flexed at 90 degrees (@90) and with the knee straight (@180). Missing data is specified with “X”. AFO category is given as “FES” (Functional Electrical Stimulation), “Articulated” (where ankle movement occurs through a built-in mechanical joint), “Flexible” (where some ankle movement is allowed due to flexibility in the material) and “Rigid” (no ankle movement is allowed). The amount movement available in each AFO is given separately for plantarflexion and dorsiflexion as “free” (motion is not restricted), “some” (the flexibility in the material or mechanical joint imposes limits to motion but does not prevent all motion) and “none”(no ankle movement is available).

Under materials/components, the materials and joints which define the stiffness of each AFO is given. For prefabricated AFOs, the model’s name and manufacturer is provided. Because FES consists of a cuff below the knee, no material or component is placed around the ankle.

Participant	Gait Pattern	Age (years)	CP distribution	GMFCS	Ankle ROM of most affected side in degrees (knee@90)	(knee@180)	AFO category	Plantarflexion available in AFO	Dorsiflexion available in AFO	Materials/components
1	Mild	15	Left	1	15	10	FES	free	free	-
2	Mild	6	Left	1	0	0	Flexible	some	some	Carbon Fibre Prepreg (ToeOff®, Allard)
3	Dropfoot	11	Right	1	10	2	Articulated	none	free	Thermoplastic (with Tamarack® Flexure Joint, Becker Orthopedic)
4	Dropfoot	9	Left	1	15	20	Articulated	none	free	Thermoplastic (with Tamarack® Flexure Joint, Becker Orthopedic)
5	Dropfoot	9	Right	1	20	10	Articulated	none	free	Thermoplastic (with Tamarack® Flexure Joint, Becker Orthopedic)
6	Dropfoot	15	Right	2	0	0	Flexible	some	some	Carbon Fibre Prepreg (WalkOn®, OttoBock)
7	Dropfoot	8	Right	1	15	0	Flexible	some	some	Carbon Fibre Prepreg (ToeOff®, Allard)
8	Dropfoot	14	Left	2	0	0	Flexible	some	some	Carbon Fibre & PEEK (Dynamic Walk®, Fillauer)
9	Dropfoot	9	Left	1	10	0	Flexible	none	free	Thermoplastic (with Tamarack® Flexure Joint, Becker Orthopedic)

Participant	Gait Pattern	Age (years)	CP distribution	Participant	Ankle ROM of most affected side in degrees (knee@90)	(knee@180)	AFO category	Plantarflexion available in AFO	Dorsiflexion available in AFO	Materials/components
10	Dropfoot	12	Left	1	5	5	Flexible	some	some	Carbon Fibre Prepreg (ToeOff®, Allard)
11	Dropfoot	10	Right	1	20	10	FES	free	free	-
12	Dropfoot	9	Right	1	10	0	Articulated	**none**	free	Thermoplastic (with Tamarack® Flexure Joint, Becker Orthopedic)
13	Dropfoot	7	Bilateral	2	10	5	Flexible	some	some	Carbon Fibre Prepreg (ToeOff®, Allard)
14	Dropfoot	14	Bilateral	1	X	X	Flexible	some	some	Carbon Fibre Prepreg (ToeOff®, Allard)
15	Dropfoot	18	Right	2	5	0	Flexible	some	some	Carbon Fibre Prepreg (ToeOff®, Allard)
16	Dropfoot	11	Left	1	10	10	Flexible	some	some	Carbon Fibre Prepreg (ToeOff®, Allard)
17	Dropfoot	9	Right	1	10	5	Flexible	some	some	Carbon Fibre Prepreg (ToeOff®, Allard)
18	Dropfoot	8	Left	1	10	5	Flexible	some	some	Thermoplastic (posterior trimlines)
19	Dropfoot	9	Left	1	20	10	Flexible	some	some	Carbon Fibre Prepreg (ToeOff®, Allard)
20	Dropfoot	10	Left	1	15	5	Flexible	some	some	Carbon Fibre Prepreg (ToeOff®, Allard)
21	Genu Recurvatum	13	Left	1	5	10	Articulated	**none**	free	Thermoplastic (with Tamarack® Flexure Joint, Becker Orthopedic)
22	Genu Recurvatum	12	Right	2	10	10	Articulated	**none**	free	Thermoplastic (with Tamarack® Flexure Joint, Becker Orthopedic)
23	Genu Recurvatum	6	Left	2	30	20	Articulated	**none**	free	Thermoplastic (with Tamarack® Flexure Joint, Becker Orthopedic)
24	Genu Recurvatum	6	Left	1	5	0	Articulated	**none**	free	Thermoplastic (with Tamarack® Flexure Joint, Becker Orthopedic)

Participant	Gait Pattern	Age (years)	CP distribution	Participant	Ankle ROM of most affected side in degrees (knee@90)	(knee@180)	AFO category	Plantarflexion available in AFO	Dorsiflexion available in AFO	Materials/components
25	Genu Recurvatum	9	Left	1	20	10	Articulated	**none**	free	Thermoplastic (with Tamarack® Flexure Joint, Becker Orthopedic)
26	Genu Recurvatum	6	Bilateral	1	20	5	Flexible	some	some	Carbon Fibre Prepreg (Kiddie Gait®, Allard)
27	Genu Recurvatum	12	Left	1	5	0	Articulated	**none**	free	Thermoplastic (with Tamarack® Flexure Joint, Becker Orthopedic)
28	Genu Recurvatum	16	Bilateral	2	0	0	Flexible	some	some	Carbon Fibre Prepreg (ToeOff®, Allard)
29	Genu Recurvatum	16	Left	2	5	0	FES	free	free	-
30	Genu Recurvatum	10	Left	1	15	5	Articulated	**none**	free	Thermoplastic (with Tamarack® Flexure Joint, Becker Orthopedic)
31	Genu Recurvatum	8	Left	1	10	10	Articulated	**none**	free	Thermoplastic (with Tamarack® Flexure Joint, Becker Orthopedic)
32	Genu Recurvatum	16	Bilateral	2	X	X	Articulated	5 deg.	5 deg.	Carbon Fibre Prepreg (ROM adjustable ankle joint)
33	Genu Recurvatum	16	Left	2	-5	-5	Flexible	some	some	Thermoplastic
34	Genu Recurvatum	8	Left	1	5	0	Flexible	some	some	Carbon Fibre Prepreg (ToeOff®, Allard)
35	Genu Recurvatum	8	Bilateral	2	20	5	Flexible	some	some	Thermoplastic (posterior trimlines)
36	Genu Recurvatum	9	Right	1	5	0	Articulated	**none**	free	Thermoplastic (with Tamarack® Flexure Joint, Becker Orthopedic)
37	Genu Recurvatum	7	Left	1	15	5	Flexible	some	some	Carbon Fibre Prepreg (ToeOff®, Allard)
38	Genu Recurvatum	14	Bilateral	1	5	5	Articulated	**none**	free	Thermoplastic (with Tamarack® Flexure Joint, Becker Orthopedic)
39	Genu Recurvatum	11	Left	1	5	0	Flexible	some	some	Carbon Fibre Prepreg (ToeOff®, Allard)

Participant	Gait Pattern	Age (years)	CP distribution	Participant	Ankle ROM of most affected side in degrees (knee@90)	(knee@180)	AFO category	Plantarflexion available in AFO	Dorsiflexion available in AFO	Materials/components
40	Genu Recurvatum	9	Bilateral	2	5	-10	Flexible	some	some	Thermoplastic (with posterior trimlines)
41	Genu Recurvatum	6	Bilateral	2	15	0	Articulated	**none**	free	Thermoplastic (with Tamarack® Flexure Joint, Becker Orthopedic)
42	True Equinus	9	Left	1	20	10	Articulated	**none**	free	Thermoplastic (with Tamarack® Flexure Joint, Becker Orthopedic)
43	True Equinus	6	Bilateral	1	20	15	Articulated	some	some	Carbon Fibre Prepreg (spring loaded, ROM adjustable)
44	Jump Gait	12	Right	1	10	10	Articulated	**none**	free	Thermoplastic (with Tamarack® Flexure Joint, Becker Orthopedic)
45	Jump Gait	8	Right	2	10	5	Articulated	some	some	Carbon Fibre Prepreg (spring loaded, ROM adjustable)
46	Jump Gait	5	Right	1	25	20	Articulated	**none**	free	Thermoplastic (with Tamarack® Flexure Joint, Becker Orthopedic)
47	Jump Gait	8	Left	1	0	0	Articulated	some	some	Carbon Fibre Prepreg (spring loaded, ROM adjustable)
48	Jump Gait	10	Bilateral	2	X	X	Articulated	**none**	free	Thermoplastic (with Tamarack® Flexure Joint, Becker Orthopedic)
49	Jump Gait	8	Right	1	5	0	Articulated	**none**	free	Thermoplastic (with Tamarack® Flexure Joint, Becker Orthopedic)
50	Jump Gait	6	Left	1	0	0	Flexible	some	some	Carbon Fibre Prepreg (ToeOff®, Allard)
51	Apparent Equinus	6	Left	1	5	5	Rigid AFO	**none**	**none**	Thermoplastic (anterior trimlines)
52	Apparent Equinus	7	Left	1	20	20	Articulated	**none**	free	Thermoplastic (with Tamarack® Flexure Joint, Becker Orthopedic)
53	Apparent Equinus	6	Right	1	X	X	Articulated	**none**	free	Thermoplastic (with Tamarack® Flexure Joint, Becker Orthopedic)
54	Crouch Gait	10	Left	2	25	15	Articulated	**none**	free	Thermoplastic (with Tamarack® Flexure Joint, Becker Orthopedic)

Participant	Gait Pattern	Age (years)	CP distribution	Participant	Ankle ROM of most affected side in degrees		AFO category	Plantarflexion available in AFO	Dorsiflexion available in AFO	Materials/components
					(knee@90)	(knee@180)				
55	Crouch Gait	13	Left	2	5	5	Flexible	some	some	Carbon Fibre Prepreg (ToeOff®, Allard)
56	Crouch Gait	16	Bilateral	2	X	X	Flexible	some	some	Carbon Fibre Prepreg (ToeOff®, Allard)
57	Crouch Gait	18	Bilateral	2	10	0	Rigid AFO	**none**	**none**	Carbon Fibre Prepreg
58	Crouch Gait	6	Bilateral	2	25	20	Flexible	some	some	Carbon Fibre Prepreg (Kiddie Gait, Allard)
59	Crouch Gait	7	Bilateral	2	20	5	Articulated	**none**	free	Thermoplastic (with Tamarack® Flexure Joint, Becker Orthopedic)
60	Crouch Gait	7	Right	1	5	5	Articulated	**none**	free	Thermoplastic (with Tamarack® Flexure Joint, Becker Orthopedic)
61	Crouch Gait	14	Bilateral	2	0	-5	Flexible	some	some	Carbon Fibre Prepreg (ToeOff®, Allard)
62	Crouch Gait	10	Bilateral	2	20	10	Articulated	**none**	free	Thermoplastic (with Tamarack® Flexure Joint, Becker Orthopedic)
63	Crouch Gait	17	Left	1	20	20	Flexible	some	some	Carbon Fibre Prepreg (ToeOff®, Allard)
64	Crouch Gait	8	Right	2	30	15	Flexible	some	some	Carbon Fibre Prepreg (ToeOff®, Allard)
65	Crouch Gait	10	Bilateral	2	10	10	Articulated	some	some	Carbon Fibre Prepreg (spring loaded, ROM adjustable)
66	Not classifiable	12	Left	1	30	25	Flexible	some	some	Carbon Fibre Prepreg (ToeOff®, Allard)
